# Molecular and biological characterization of *Chilli leaf curl virus* and associated *Tomato leaf curl betasatellite* infecting tobacco in Oman

**DOI:** 10.1186/s12985-019-1235-4

**Published:** 2019-11-09

**Authors:** Muhammad Shafiq Shahid, Muhammad Shafiq, Amir Raza, Abdullah M. Al-Sadi, Rob W. Briddon

**Affiliations:** 1 Department of Crop Sciences, College of Agricultural and Marine Sciences, Sultan Qaboos University, Al-Khod, 123 Muscat, Oman; 2 Agricultural Biotechnology Division, National Institute for Biotechnology and Genetic Engineering, Faisalabad, Pakistan

**Keywords:** Geminivirus, Begomovirus, Betasatellite, *Chilli leaf curl virus*, *Tomato leaf curl betasatellite*, *Nicotiana tabacum*

## Abstract

**Background:**

In Oman tobacco (*Nicotiana tabacum*; family Solanaceae) is a minor crop, which is produced only for local consumption. In 2015, tobacco plants exhibiting severe downward leaf curling, leaf thickening, vein swelling, yellowing and stunting were identified in fields of tobacco in Suhar Al-Batina region, Oman. These symptoms are suggestive of begomovirus (genus *Begomovirus*, family *Geminiviridae*) infection.

**Methods:**

Circular DNA molecules were amplified from total DNA extracted from tobacco plants by rolling circle amplification (RCA). Viral genomes were cloned from RCA products by restriction digestion and betasatellites were cloned by PCR amplification from RCA product, using universal primers. The sequences of full-length clones were obtained by Sanger sequencing and primer walking. Constructs for the infectivity of virus and betasatellite were produced and introduced into plants by *Agrobacterium*-mediated inoculation.

**Results:**

The full-length sequences of 3 begomovirus and 3 betasatellite clones, isolated from 3 plants, were obtained. Analysis of the full-length sequences determined showed the virus to be a variant of *Chilli leaf curl virus* (ChiLCV) and the betasatellite to be a variant of *Tomato leaf curl betasatellite* (ToLCB). Both the virus and the betasatellite isolated from tobacco show the greatest levels of sequence identity to isolates of ChiLCV and ToLCB identified in other hosts in Oman. Additionally clones of ChiLCV and ToLCB were shown, by *Agrobacterium*-mediated inoculation, to be infectious to 3 *Nicotiana* species, including *N. tabacum*. In *N. benthamiana* the betasatellite was shown to change the upward leaf rolling symptoms to a severe downward leaf curl, as is typical for many monopartite begomoviruses with betasatellites.

**Conclusions:**

The leaf curl disease of tobacco in Oman was shown to be caused by ChiLCV and ToLCB. This is the first identification of ChiLCV with ToLCB infecting tobacco. The study shows that, despite the low diversity of begomoviruses and betasatellites in Oman, the extant viruses/betasatellites are able to fill the niches that present themselves.

## Background

Viruses of the genus *Begomovirus* (family *Geminiviridae*) cause economically important diseases of many crops throughout tropical and subtropical regions. Begomoviruses have circular single-stranded (ss) DNA genomes, that are encapsidated in distinctive twinned icosahedral (geminate) particles and are transmitted exclusively by the whitefly *Bemisia tabaci* [1]. The genomes of begomoviruses are either bipartite, consisting of two ~ 2.6–2.8 kb genomic components known as DNA A and DNA B, or monopartite, consisting of a single ~ 2.6–2.8 kb component that is a homolog of the DNA A of bipartite viruses. The majority of begomoviruses native to the New World are bipartite, whereas the majority of begomoviruses native to the Old World (OW) are monopartite [1]. The genomes (or DNA A components) of begomoviruses originating from the OW encode six genes. In the complementary-sense the genes encode the replication-associated protein, the transcriptional-activator protein, the replication-enhancer protein and the (A) C4 protein. The two genes in the virion-sense encode the (A) V2 protein and the coat protein [2].

Most monopartite begomoviruses are associated with a group of ssDNA satellites collectively known as betasatellites [3, 4]. Betasatellites are approximately half the size of their helper begomoviruses (~ 1.4 kb) and depend on the helper virus for their replication, movement in plants and transmission between plants [4]. The structure of betasatellites is highly conserved comprising of a sequence rich in adenine (A-rich), a sequence conserved between all betasatellites, known as the satellite conserved region (SCR), that contains a predicted hairpin structure with the nonanucleotide sequence TAATATTAC forming part of the loop, and a single conserved (between all betasatellites) gene with a capacity to encode an ≥118 amino acid product known as βC1 [5].

The first disease caused by a begomovirus in Oman was identified in 1993, although the causative viruses and satellites were not characterized until much later [6]. Despite this the identified diversity of begomoviruses and betasatellites in Oman remains low relative to the known diversity on the Indian sub-continent. The majority of these viruses and satellites appear to have been introduced to the country or have evolved from introduced species. In this respect Oman holds an unusual position, being at the front of a meeting of begomoviruses introduced from Africa, such as *Tomato leaf curl Sudan virus* and *African cassava mosaic Zanzibar virus* [7, 8], begomoviruses and satellites introduced from the Indian sub-continent, such as *Chilli leaf curl viru*s (ChiLCV) and *Tomato leaf curl betasatellite* (ToLCB [9];), and viruses native to the Middle East region, such as *Tomato yellow leaf curl virus* (TYLCV) and *Watermelon chlorotic stunt virus* [10, 11].

The study described here has analysed the etiology of a leaf curl disease of tobacco recently identified for the first time in Oman. The results show that the disease is associated with ChiLCV and ToLCB. Additionally *Agrobacterium*-mediated inoculation of the cloned virus and betasatellite were used to satisfy Koch’s postulates. The significance of the findings is discussed.

## Methods

### DNA extraction and initial detection of a begomovirus and satellite by polymerase chain reaction

Total nucleic acid was extracted from leaf samples using a cetyltrimethylammonium bromide-based method [12] and kept at − 20 °C. Extracted DNA was used as a template in polymerase chain reaction (PCR) with primer pairs for the detection of begomoviruses (TYLCD-356 (5′-ATCATTTCCACKCCCGYCTCGA-3′/TYLCD-1044 5′-GCRTGMGTACABGCCATATACA-3′), amplifying an ~ 800 nt product, betasatellites (Sat101/Sat102), amplifying an ~ 1350 nt product [13], and alphasatellites (DNA101/DNA102), amplifying an ~ 1380 nt product [14]. Additionally the primer pair βC1F (5′-AGACCCGGGATGACGATCAGATATAATAACA-3′)/βC1R (5′-ACGTCGACTCACACACACACTTTCGTACA-3′), amplifying a ~ 350 nt product, was used in PCR for the detection of betasatellites.

### Rolling circle amplification, cloning and sequencing

Circular DNA molecules in nucleic acid samples were enriched using rolling circle amplification (RCA) as described earlier [15]. Restriction of the high molecular weight concatameric RCA products with *Bam*HI resulted in ~ 2.7 kb fragments, which were eluted from agarose gels using a GeneJet Gel Extraction Kit (Thermo Fisher Scientific) and cloned in *Bam*HI restricted pGEM-3zf (+) (Promega Madison, USA). Potentially full-length clones resulting from RCA (for begomovirus) and PCR (for betasatellite) were sequenced commercially using the primer-walking approach (Macrogen Inc., South Korea).

### Sequence assembly and analysis

Sequences were assembled using SeqMan, part of the Lasergene package of sequence analysis software (DNA Star Inc., Madison, WI, USA). Related sequences available in the GenBank database were identified using the Basic Local Alignment Search Tool nucleotide (BLASTn) [16] run on-line (http://blast.ncbi.nlm.nih.gov/Blast.cgi). Open reading frames (ORFs) in sequences were identified using the ORF Finder program run on-line (https://www.ncbi.nlm.nih.gov/orffinder/). The Species Demarcation Tool (SDT), with the MUSCLE option, was used to calculate sequence identity values [17]. Pairwise multiple sequence alignments were produced using the MUSCLE algorithm implemented in MEGA6 [18]. The evolutionary relationships between sequences were determined by constructing phylogenetic trees using Clustal X (neighbor-joining method) and displayed using Treeview [19].

### Production of constructs for *Agrobacterium*-mediated inoculation of plants

Clone Tob11was digested with *Hin*dIII and *Sal*I to release a fragment of ~ 1000 bp which was ligated into the binary vector pGreen0029 [20]. Then the full-length insert of Tob11, released using *Hin*dIII, was ligated into the pGreen0029 clone containing the fragment, linearized using *Hin*dIII, to yield a partial direct repeat of the virus genome. A similar strategy was used to produce a construct for clone Tob44 in pGreen0029 using a ~ 425 bp *Kpn*I-*Xba*I fragment and the full-length insert released using *Kpn*I.

### *Agrobacterium*-mediated inoculation of plants

Binary vector constructs were electroporated into *Agrobacterium tumefaciens* strain LBA4404. *Agrobacterium* inocula were prepared and inoculated to *Nicotiana benthamiana*, *N. glutinosa* and *N. tabacum* seedlings by agroinfiltration as described previously [21]. Infiltrated plants were maintained in an insect-free growth room at 28 °C with a photoperiod of 16 h and monitored daily for the appearance of symptoms.

### Southern blot hybridization

Total genomic DNA (~ 10 μg) extracted from plant tissue was resolved on 1.5% agarose gels, transferred overnight by capillary action onto Hybond N^+^ membrane (Amersham) and UV cross-linked. Probes were produced by PCR using a DIG-high prime kit (Roche Applied Science) and primers βC1F (5′-AGACCCGGGATGACGATCAGATATAATAACA-3′)/ βC1R (ACGTCGACTCACACACACACTTTCGTACA-3′) for betasatellite and (qPCRChV F (5′-AGTTCATATAGGGAAGGTTATGTGTA-3′) / qPCRChV R (5′- GGACAAGGAAGAACATCACACTATTC-3′) for virus. Membranes were hybridized and washed as described previously [22, 23] and hybridization was detected by colorimetric detection with nitro blue tetrazolium chloride and 5-bromo-4-chloro-3-indolyl-phosphate, toluidine-salt (Roche Applied Science).

## Results

### Identification and characterization of a begomovirus and betasatellite infecting tobacco in Oman

**Fig. 1:**
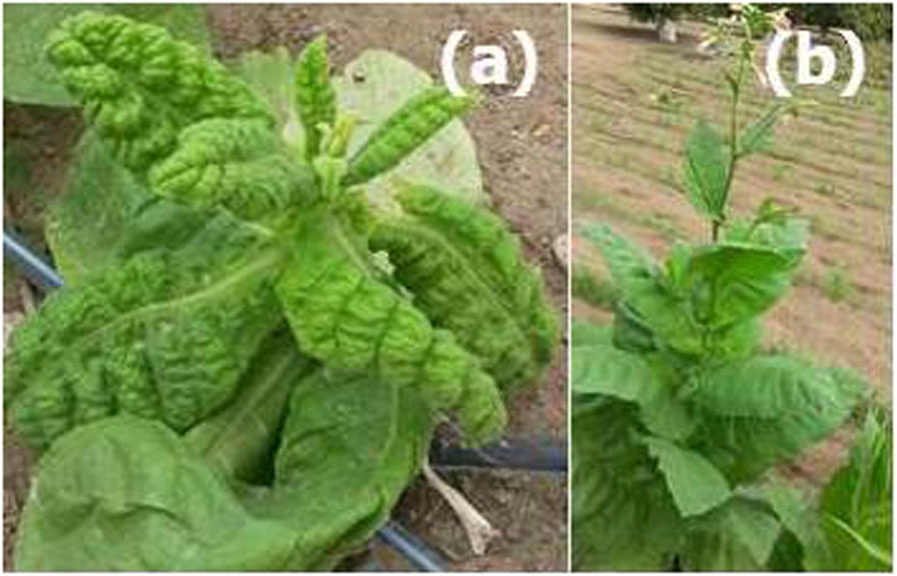
Tobacco plant exhibiting severe downward leaf curling, leaf thickening, vein swelling, yellowing and stunting symptoms (**a**) compared to a non-symptomatic tobacco plant (**b**) growing in the same field

During a survey for virus-infected plants in December 2015 on an organic farm in Sohar (Al Batinah region, Sultanate of Oman), fields of tobacco (*Nicotiana tabacum*) were observed with plants showing severe downward leaf curling, leaf thickening, vein swelling, mild yellowing and stunting (Fig. 1). In the fields between 60 and 70% of plants were exhibiting symptoms.

**Fig. 2:**
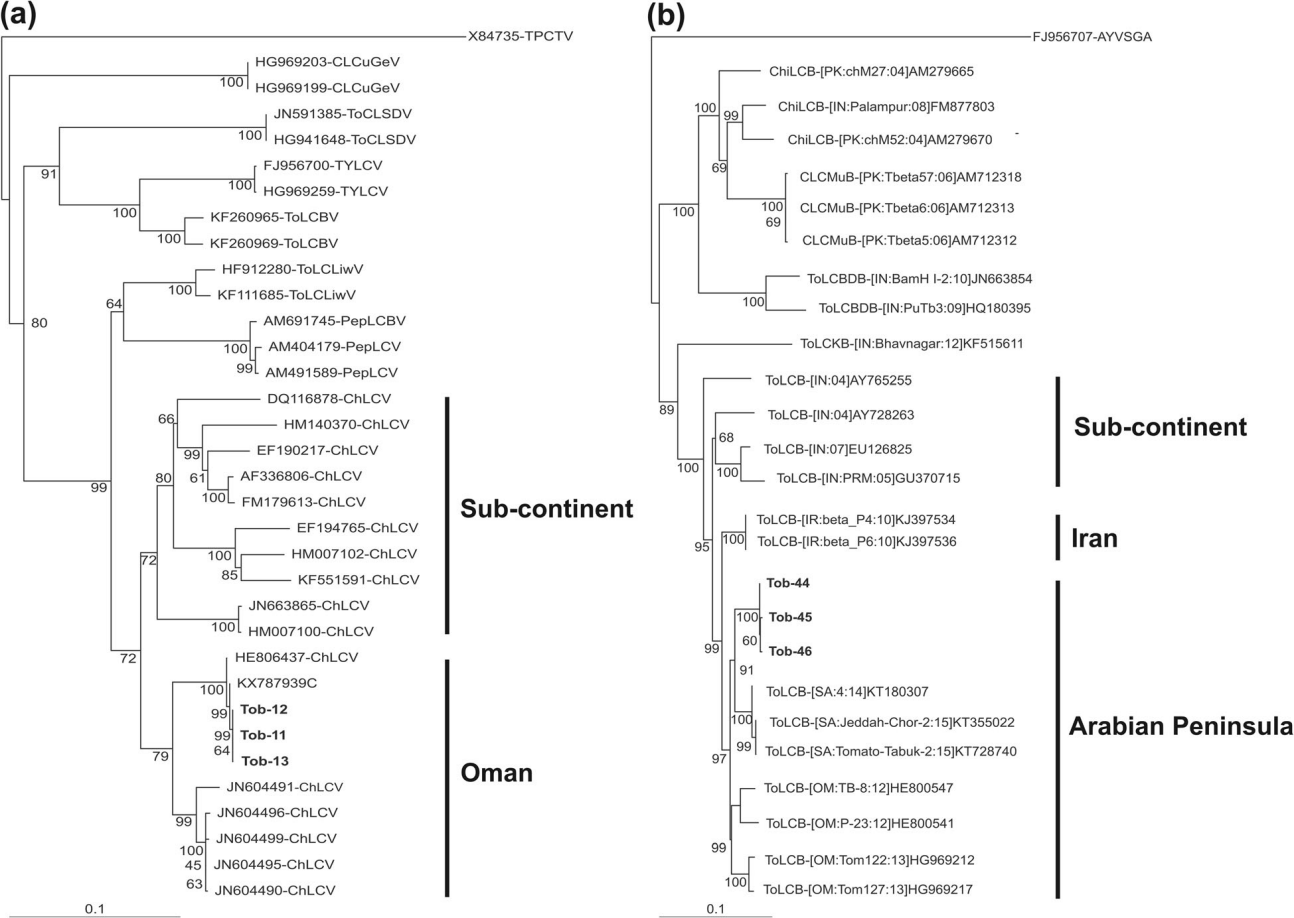
Phylogenetic dendrograms based upon alignments of the complete nucleotide sequences of the ChiLCV (**a**) or ToLCB (**b**) clones obtained from tobacco in Oman with selected begomovirus and betasatellite sequences obtained from the databases. Vertical branches are arbitrary, horizontal branches are proportional to calculated mutation distance. Values at nodes indicate percentage boot strap values (1000 replicates). Begomovirus acronyms used are *Chilli leaf curl virus* (ChLCV), *Cotton leaf curl Gezira virus* (CLCuGeV), *Pepper leaf curl virus* (PepLCV), *Tomato leaf curl Liwa virus* (ToLCLwV), *Tomato leaf curl Sudan virus* (ToLCSDV) and *Tomato yellow leaf curl virus* (TYLCV). The betasatellite acronyms used are *Chilli leaf curl betasatellite* (ChiLCB), *Cotton leaf curl Multan betasatellite* (CLCuMuB), *Tomato leaf curl Bangladesh betasatellite* (ToLCBDB), *Tomato leaf curl betasatellite* (ToLCB) and *Tomato leaf curl Karnataka betasatellite* (ToLCKB). The trees were arbitrarily rooted on the sequence *Tomato pseudo-curly top virus* (TPCTV; X84735), for the virus tree, and *Ageratum yellow vein Singapore alphasatellite* (AYVSGA; FJ956707), for the betasatellite tree, as outgroup. The database accession numbers are indicated in each case. The sequences originating from tobacco are indicated by bold text in each case. The geographical origins of ChiLCV and ToLCB sequences are indicated on the right in each case

Leaves from four symptomatic *N. tabacum* plants, originating from two independent but neighbouring fields (500 m apart), were collected, as well as a non-symptomatic plant from each field. A high molecular weight product was obtained by RCA with DNA extracted from all four symptomatic plants but not from the non-symptomatic plants (results not shown). Three potentially full-length clones (Tob11, Tob12 and Tob13), resulting from digestion of RCA product with *Bam*HI, were obtained, one from each of three *N. tabacum* plants (Table 1). The sequence of each clone was 2761 nucleotide (nt) in length and are available in the nucleotide sequence databases under the accession numbers given in Table 1. Analysis of sequences showed them to have an organization of genes typical of the genomes of monopartite begomoviruses originating from the OW, with two predicted genes in the virion-sense and four in the complementary-sense (Table 1). SDT analysis showed the three sequences to have greater than 99% nucleotide sequence identity, showing them to be isolates of a single begomovirus species, based on current guidelines for species demarcation [24]. Comparison of the sequences to sequences available in the GenBank database using BLASTn showed them to have high levels of identity to isolates of the monopartite ChiLCV. Pairwise sequence analysis using SDT, and selected sequences from the database, showed the isolates from tobacco have the highest levels of sequence identity (99.9 to 100%) to an isolate of ChiLCV recently identified in watermelon in Oman (KX787939 [25];). This was supported by a phylogenetic analysis based upon an alignment of the full-length sequences of ChiLCV obtained from tobacco and selected sequences from the databases (Fig. 2a). Isolates of ChiLCV originating from Oman form a clade distinct from the ChiLCV isolates originating from the Indian sub-continent, with the sequences from tobacco being most closely related to the ChiLCV isolate from watermelon.

**Table 1:** Percentage identity of *Chilli leaf curl virus* and *Tomato leaf curl betasatellite* isolated from tobacco to selected other begomoviruses and betasatellites

ChiLCV (Tob11)					ToLCB (Tob44)				
Accession	Virus Acronym^a^	Host species	Country	Percentage identity	Accession	Betasatellite Acronym^a^	Host species	Country	Percentage identity
KX787939	ChiLCV	*Citrullus lanatus*	Oman	100	KT180307	ToLCB	Cucumber	Saudi Arabia	95.6
JQ654463	ChiLCBV	*Amaranthus viridis*	India	81.1	KT355022	ToLCB	*Corchorus*	Saudi Arabia	96.3
JQ765395	ToLCJV	*Gaillardia* spp.	India	77.4	MG571547	ToLCB	*Mentha*	Saudi Arabia	95.8
KX671562	PeLCV	*Glycine max*	Pakistan	76	KJ397536	ToLCB	Papaya	Iran	94.2
LM645009	HoLCV	Okra	Pakistan	76.6	KF515611	ToLCKaB	Tomato	India	85.2
AM491589	PepLCV	*Capsicum annuum*	Pakistan	75.8	AM712312	CLCuMB	*Gossypium anomalum*	Pakistan	81.9
AM691745	PepLCBV	*Capsicum annum*	Pakistan	75.4	AY438558	ToLCRaB	Tomato	Bangladesh	81.0
KF260965	ToLCBV	Tomato	Oman	74.8	HQ180395	ToLCBDB	*Nicotiana tabacum*	India	81.0
FJ956700	TYLCV	Tomato	Oman	70.5	AY244706	PaLCuB	Papaya	India	69.0
JN591385	ToLCSDV	Tomato	Oman	63.6	AM410551	CroYVMB	Croton	Pakistan	65.9
HE862273	CLCuGeV	Tomato	Oman	65.3	KF267444	OLCuB	Okra	Oman	28.3

^a^The virus acronyms are given as *BYVV* Bhendi yellow vein virus, *ChiLCV* Chilli leaf curl virus, *CLCuGeV* Cotton leaf curl Gezira virus, *HoLCV* Hollyhock leaf curl virus, *PeLCV* Pedilanthus leaf curl virus, *PepLCBV* Pepper leaf curl Bangladesh virus, *ToLCJV* Tomato leaf curl Joydebpur virus , *ToLCSDV* Tomato leaf curl Sudan virus and *TYLCV* Tomato yellow leaf curl virus. The betasatellite acronyms are given as *CLCuMuB* Cotton leaf curl Multan betasatellite, *CroYVMB* Croton yellow vein mosaic betasatellite, *OLCuB* Okra leaf curl betasatellite, *PaLCuB* Papaya leaf curl betasatellite, *ToLCBDB* Tomato leaf curl Bangladesh betasatellite, *ToLCB* Tomato leaf curl betasatellite, *ToLCKaB* Tomato leaf curl Karnataka betasatellite and *ToLCRaB* Tomato leaf curl Ranchi betasatellite

Three clones were obtained by PCR with betasatellite primers β101/102, one each from the three plants from which the virus clones were obtained (Additional file 1: Table S1). The sequences are 1375 (clone Tob45) and 1377 nt (clones Tob44 and Tob46) in length and are available in the nucleotide sequence databases under the accession numbers given in Additional file 1: Table S1. Analysis of the sequences showed them to have an organization and features typical of betasatellites [5], consisting a single conserved (between all betasatellites) ORF in the complementary-sense (known as βC1) having the capacity to encode a product of 118 amino acid, a region of sequence rich in adenine (coordinates 722–968 nt) and a sequence well conserved between all betasatellites (known as the satellite conserved region; coordinates 1252–21 nt) that contains a predicted stem-loop structure with, as part of the loop, the nonanucleotide sequence TAATATTAC with similarity to the origin of replication of geminiviruses.

SDT analysis showed the three betasatellites isolated from tobacco to have between 99.7 and 99.8% nucleotide sequence identity with each other. Comparison of the three sequences from tobacco to sequences available in the GenBank database using BLASTn showed them to have high levels of nucleotide sequence identity to isolates of the betasatellite ToLCB. Pairwise sequence analysis using SDT, and selected ToLCB sequences from the databases, showed the isolates from tobacco have the highest levels of sequence identity (99.9%) to an isolate of ToLCB recently identified in watermelon in Oman (KX787940 [25];). Overall the sequences of ToLCB from tobacco showed higher levels of sequence identity to isolates of ToLCB originating from the Arabian Peninsula than to isolates either from Iran or the sub-continent. This is supported by a phylogenetic analysis (Fig. 2b) which shows ToLCB isolates from the Arabian Peninsula, including those identified here in tobacco, form a clade separate from other ToLCB isolates but to be most closely related to isolates from Iran.

### Infectivity of cloned ChiLCV and ToLCB

**Fig. 3:**
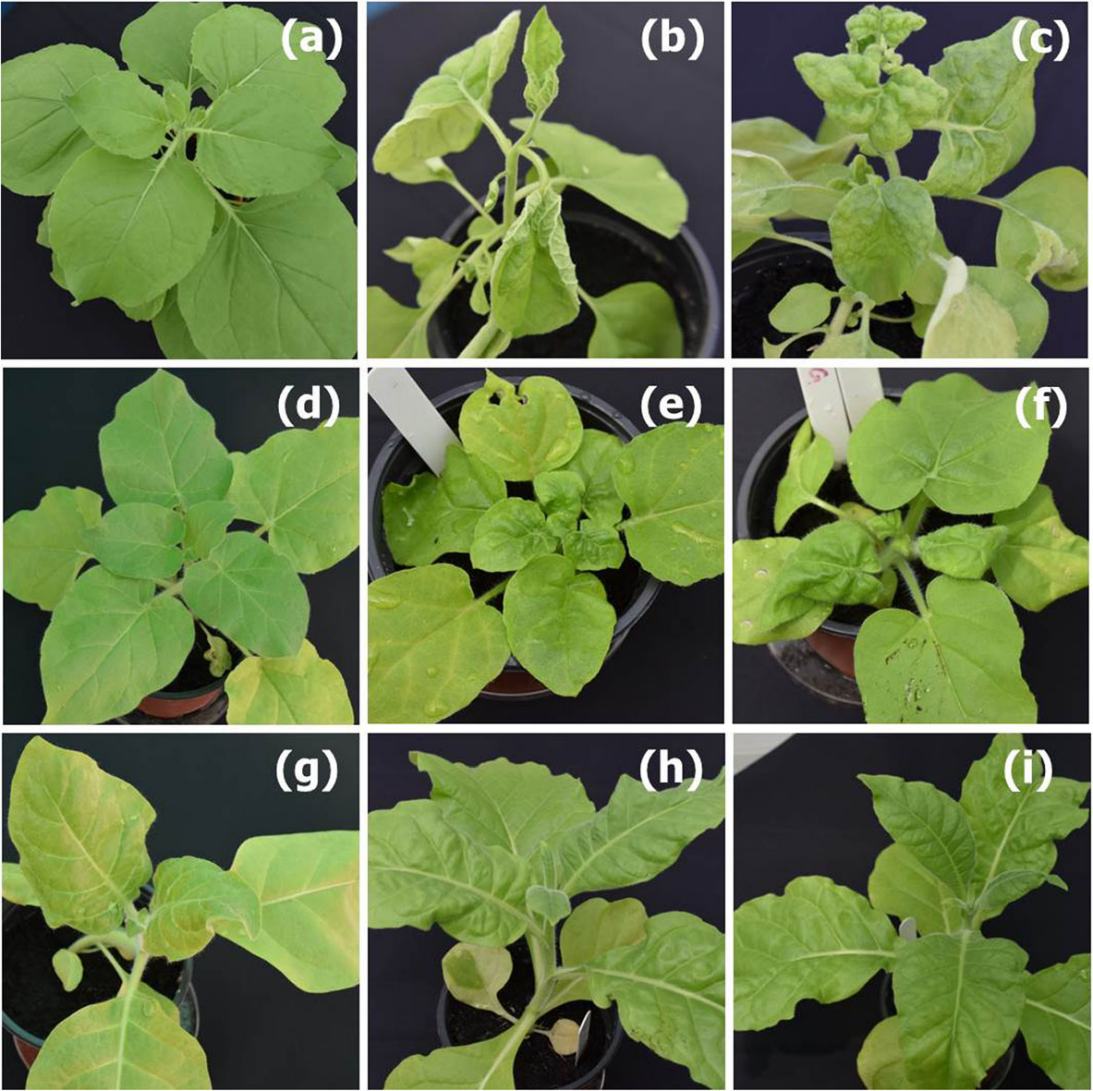
Symptoms induced in three plant species by *Agrobacterium*-mediated inoculation with ChiLCV and ToLCB. Shown are *Nicotiana benthamiana* (**a**-**c**), *N. glutinosa* (**d**-**f**) and *N. tabacum* (**g**-**i**) plants either mock inoculated with an *Agrobacterium* culture harbouring an empty binary vector (**a**, **d**, **g**), inoculated with an *Agrobacterium* culture harbouring a binary vector construct for the infectivity of ChiLCV (**b**, **e**, **h**), or co-inoculated with *Agrobacterium* cultures harbouring binary vector constructs for the infectivity of ChiLCV and ToLCB (**c**, **f**, **i**). Plants were photographed at 24 days after inoculation

To satisfy Koch’s postulates a partial direct repeat construct of ChiLCV was inoculated to *Nicotiana benthamiana*, *N. glutinosa* and *N. tabacum* plants, either in the presence of absence of a construct for the associated ToLCB (Table 2; Fig. 3). Inoculation of just the ChiLCV construct to *N. benthamiana* seedlings resulted in severe upward leaf curling and vein swelling on the undersides of young, newly developing leaves initiating at 16 days post-inoculation. Co-inoculation of *N. benthamiana* seedlings with the constructs for both ChiLCV and ToLCB resulted in severe downward leaf curling and crumpling young, newly developing leaves at 12 dpi.

**Table 2:** Infectivity of *Chilli leaf curl virus* and associated *Tomato leaf curl betasatellite* by *Agrobacterium*-mediated inoculation

Plant	ChLCV					ChLCV and ToLCB				
	Plants symptomatic/plants inoculated			Symptoms^a^	Latent period^b^ (dpi)	Plants symptomatic /plants inoculated			Symptoms^a^	Latent Period^b^ (dpi)
	Exp. 1	Exp. 2	Exp. 3			Exp. 1	Exp. 2	Exp. 3		
*N. benthamiana*	8/8	7/8	8/8	sulr, vs	16	8/8	8/8	8/8	sdlc, cr	12
*N. glutinosa*	7/8	8/8	6/8	cr	21	8/8	7/8	8/8	sdlc, cr	18
*N. tabacum*	5/8	4/8	3/8	mcr,	21	6/8	4/8	5/8	mdlr, mcr	18

^a^Symptoms are denoted as *cr* leaf crumpling, *mcr* mild leaf crumpling, *sdlc* severe downward leaf curl, *mdlr* mild downward leaf roll, *sulr* severe upward leaf roll and *vs* foliar vein swelling

^b^Time between inoculation and first appearance of symptoms

Inoculation of *N. glutinosa* with only the ChiLCV construct resulted in leaf crumpling which initiated at 21 dpi. As was the case for *N. benthamiana*, co-inoculation of *N. glutinosa* with the construct for ToLCB resulted in symptoms which appeared earlier (18 dpi) and the symptoms showed a pronounced downward leaf curling. Similarly for *N. tabacum* inclusion of ToLCB in the inoculum led to a decrease in latent period (from 21 to 18 dpi) and an increase in symptom severity, with slightly more severe downward leaf curling, although this was less pronounced than for either *N. benthamiana* or *N. glutinosa*. However, for both singly inoculated plants and co-inoculated plants, the infectivity was significantly less to *N. tabacum* (50 and 60%, respectively) than to either of the other *Nicotiana* species.

**Fig. 4:**
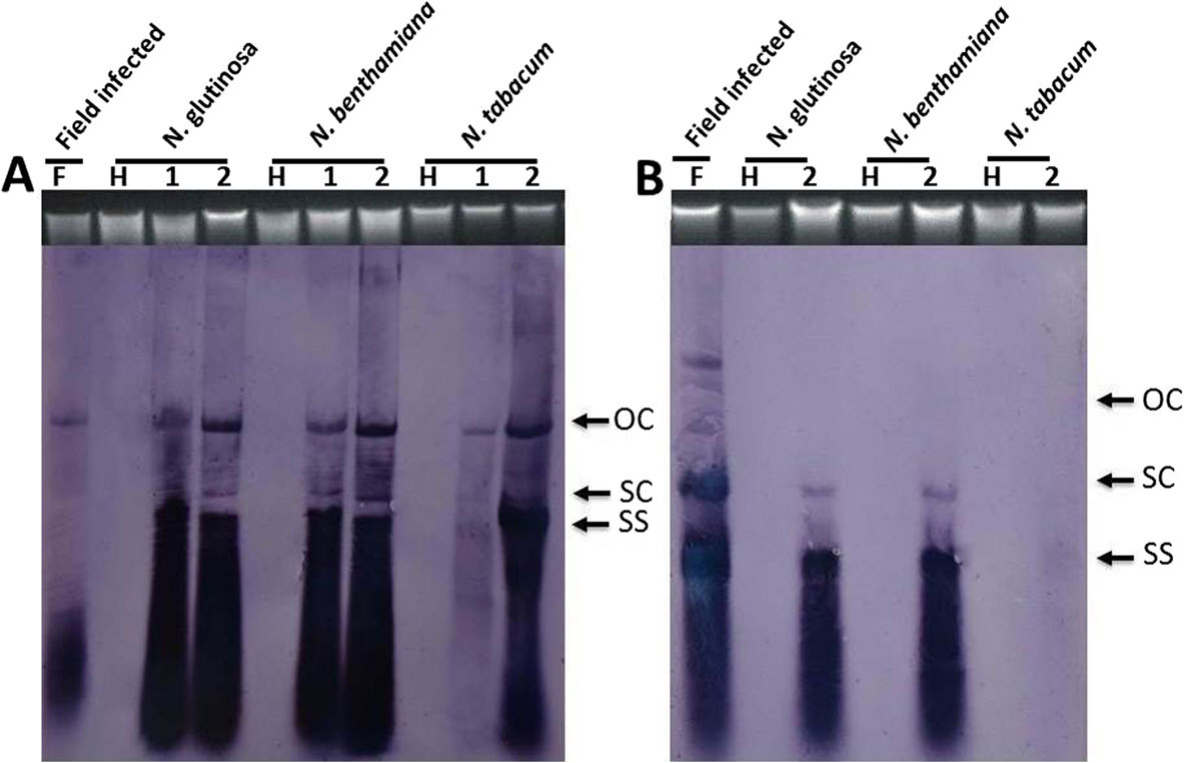
Southern blot analysis of DNA extracted from agroinoculated *N. tabacum* plants. Blots were probed for the presence of ChiLCV (**a**) and ToLCB (**b**). The samples run on the gels were extracted from *Nicotiana glutinosa*, *N. benthamiana*, and *N. tabacum* plants (as indicated) inoculated with only ChiLCV (lane 1 panel A) or co-inoculated with ChiLCV and ToLCB (lane 2 in panels A and B). In addition DNA extracted from one of the field-infected tobacco plants (F) and from a healthy, non-inoculated tobacco plant (H) was included as controls. In each case approx. 10 μg of DNA was loaded. For each blot a photograph of the ethidium bromide-stained genomic DNA band on the gel is shown to confirm equal loading. The positions of viral/betasatellite single-stranded (ss), supercoiled (sc) and open-circular DNA forms are indicated

The presence of either ChiLCV, or ChiLCV and ToLCB, was confirmed in all symptomatic inoculated plants by diagnostic PCR, whereas neither component was identified in non-inoculated and non-symptomatic inoculated plants. Southern blot analysis of DNA samples extracted from inoculated plants showed the presence of large amounts of viral DNA in both singly and co-inoculated plants and high amounts of betasatellite DNA for co-inoculated plants for *N. benthamiana* and *N. glutinosa* (Fig. 4). However, for *N. tabacum*, although there was a large amount of viral DNA detected for the plant inoculated with ChiLCV alone, much less was detected in the plant co-inoculated with ChiLCV and ToLCB. Nevertheless, the amount of viral DNA detected was comparable to the amount of viral DNA detected in field infected tobacco plant, although the amount of betasatellite detected was significantly less. For all inoculated plants, as well as the field collected plant, there was a pronounced smear of small molecular weight material below the viral and betasatellite ssDNA forms.

## Discussion

In Oman tobacco is a minor crop, grown on a small scale and only for local consumption. The results obtained here show that leaf curl symptoms in tobacco in Oman are caused by ChiLCV in association with the betasatellite ToLCB.

Only two betasatellites have been identified in Oman so far identified; ToLCB and *Okra leaf curl Oman betasatellite* [26]. ToLCB is believed to have its origins on the Indian sub-continent and to have been introduced to Oman from there. The results shown here suggest that, rather than being a direct introduction from the sub-continent, ToLCB was instead introduced into Oman indirectly, via Iran. The phylogenetic analysis certainly supports this idea, as well as the subsequent onward spread of this betasatellite across other parts of the Arabian Peninsula [27]. Possibly as a consequence of the low levels of diversity in betasatellites, ToLCB has become associated with a number of begomoviruses and causing disease a number of crops in Oman including, most recently, with the bipartite begomovirus *Mungbean yellow mosaic India virus* in bean [15]. The association of ChiLCV with ToLCB has also been shown to cause diseases of watermelon, chilli pepper and tomato in Oman [9, 25].

In common with ToLCB, ChiLCV has its origins on the sub-continent and is thought to have been introduced to Oman from there [9, 25]. In contrast to ToLCB, however, the virus has not so far been identified in Iran, so the possible route of introduction is a little less clear.

The symptoms induced by *N. benthamiana* by ChiLCV in the presence and absence very much resemble the symptoms induced in this host by *Ageratum yellow vein virus* in the presence and absence of its cognate betasatellite, *Ageratum yellow vein betasatellite* [28]. The change in symptoms in *N. benthamiana*, from an upward leaf roll to a severe downward leaf curl, has been shown to be due to betasatellites encoding a dominant symptom determinant (pathogenicity determinant) and has been shown previously for infection of *N. benthamiana* with ChiLCV and the betasatellite *Tomato leaf curl Bangladesh betasatellite* (ToLCBDB) and another Oman isolate of ChiLCV with ToLCB [9, 30]. Although Singh et al. [29] showed ToLCBDB to enhance the levels of ChiLCV DNA in *N. benthamiana*, the results of Khan et al. [9] mirrored the results obtained here; no enhancement of viral DNA levels by ToLCB. This suggests that either the virus isolates differ in their responses to betasatellites or the two betasatellites differ in their interaction with ChiLCV. This will require further investigation in the future.

Although qualitatively the symptoms induced by inoculation of *N. tabacum* with cloned ChiLCV and ToLCB resemble those seen in field infected plants, quantitatively they do not; the symptoms in field-infected plants being more severe. There are a number of possible reasons for this. It is possible that the clones obtained from tobacco are not very pathogenic (natural variants). Alternatively the conditions in the glasshouses where the experiments were conducted, required to keep whiteflies away from the experimental plants, may not be ideal for either tobacco or virus propagation. It is interesting to note that, in both experimentally inoculated plants and field collected plants there is a smear of fast migrating, small molecular weight material which hybridizes to the virus and betasatellite probes. This likely represents defective viral/betasatellite DNA replication products which may suggest that species of *Nicotiana* are not good hosts for ChiLCV and ToLCB. This idea is supported by the relatively low virus DNA levels which are present in *N. tabacum*. Nevertheless, the results show that the leaf curl disease of tobacco in Oman is caused by ChiLCV with ToLCB.

## Conclusions

The study here is the first identification of ChiLCV infecting tobacco, as well as the first identification of ToLCB in tobacco. This virus and betasatellite do not cause disease of tobacco in their regions of geographic origin. This finding is in-line with earlier studies showing that, despite the low diversity of begomoviruses and betasatellites in Oman in comparison to the sub-continent, the extant viruses/betasatellites are able to fill the niches that present themselves. Careful monitoring will be required to assess the threat of local viruses and satellites as agriculture in Oman expands and seeks to introduce new crops and crop varieties.

## Supplementary information

**Additional file 1:** **Table S1.** Features of *Chilli leaf curl virus* and associated *Tomato leaf curl betasatellite* clones isolated from field infected tobacco.

## Data Availability

The full-length sequences determined in this study were submitted to GenBank with the accession numbers MK468694 - MK468699. The datasets and materials produced for the study are available from the corresponding author on reasonable request.

## Abbreviations

AYVSGA: *Ageratum yellow vein Singapore alphasatellite*
BLASTn: Basic Local Alignment Search Tool nucleotide
BYVV: *Bhendi yellow vein virus*
ChiLCB: *Chilli leaf curl betasatellite*
ChLCV: *Chilli leaf curl virus*
CLCuGeV: *Cotton leaf curl Gezira virus*
CLCuMuB: *Cotton leaf curl Multan betasatellite*
CroYVMB: *Croton yellow vein mosaic betasatellite*
DNA: Deoxyribonucleic acid
HoLCV: *Hollyhock leaf curl virus*
nt: nucleotides
oLCBDB: *Tomato leaf curl Bangladesh betasatellite*
OLCuB: *Okra leaf curl betasatellite*
ORF: Open reading frame
PaLCuB: *Papaya leaf curl betasatellite*
PCR: Polymerase chain reaction
PeLCV: *Pedilanthus leaf curl virus*
PepLCBDV: *Pepper leaf curl Bangladesh virus*
PepLCV: *Pepper leaf curl virus*
RCA: Rolling-circle amplification
SDT: Species Demarcation Tool
ToLCB: *Tomato leaf curl betasatellite*
ToLCJV: *Tomato leaf curl Joydebpur virus*
ToLCKB: *Tomato leaf curl Karnataka betasatellite*
ToLCLwV: *Tomato leaf curl Liwa virus*
ToLCRaB: *Tomato leaf curl Ranchi betasatellite*
ToLCSDV: *Tomato leaf curl Sudan virus*
TPCTV: *Tomato pseudo-curly top virus*
TYLCV: *Tomato yellow leaf curl virus*
