# Supplementary material for: Molecular and biological characterization of Chilli leaf curl virus and associated Tomato leaf curl betasatellite infecting tobacco in Oman
Source: Virol J. 2019 Nov 9;16:131. doi: 10.1186/s12985-019-1235-4 (PMC6842478; doi:10.1186/s12985-019-1235-4)
Supplement: Supplementary file 1 — Additional file 1: Table S1. Features of Chilli leaf curl virus and associated Tomato leaf curl betasatellite clones isolated from field infected tobacco. [file 12985_2019_1235_MOESM1_ESM.docx]

**Table S1**. Features of *Chilli leaf curl virus* and associated *Tomato leaf curl betasatellite* clones isolated from field infected tobacco.

| Plant | Begomovirus | | | | | | | | | Betasatellite | | | |
| --- | --- | --- | --- | --- | --- | --- | --- | --- | --- | --- | --- | --- | --- |
|  | Clone | Accession no. | Size (nt) | Position of genes (coordinates)/no. of amino acids [predicted coding capacity in kDa] | | | | | |  |  |  |  |
|  |  |  |  | CP | V2 | Rep | TrAP | REn | C4 | Clone | Accession no. | Size (nt) | Position of βC1 gene(coordinates)/  no. of amino acids [predicted coding capacity in kDa] |
| 1 | Tob11 | MK468694 | 2,761 | 309 – 1082/  257 (29.71) | 149 – 514/  121 (12.69) | 1531 – 2616/  361 (40.23) | 1224 – 1628/  134 (15.78) | 1079 – 1483/  134 (15.78) | 2166– 2459/  97 (11.12) | Tob44 | MK468697 | 1,377 | 201 -557/  118 (13.62) |
| 2 | Tob12 | MK468695 | 2,761 | 309 – 1082/  257 (29.71) | 149 – 514/  121 (12.69) | 1531 – 2616/  361 (40.23) | 1224 – 1628/  134 (15.78) | 1079 – 1483/  134 (15.78) | 2166– 2459/  97 (11.12) | Tob45 | MK468698 | 1,375 | 201 -557/  118 (13.62) |
| 3 | Tob13 | MK468696 | 2,761 | 309 – 1082/  257 (29.71) | 149 – 514/  121 (12.69) | 1531 – 2616/  361 (40.23) | 1224 – 1628/  134 (15.78) | 1079 – 1483/  134 (15.78) | 2166– 2459/  97 (11.12) | Tob46 | MK468699 | 1,377 | 201 -557/  118 (13.62) |
